# Improved 3D measurement with a novel preprocessing method in DFP

**DOI:** 10.1186/s40638-017-0077-z

**Published:** 2017-11-17

**Authors:** Yi Xiao, You-Fu Li

**Affiliations:** Department of Mechanical and Biomedical Engineering, City University of Hong Kong, Tat Chee Avenue, Kowloon, Hong Kong

**Keywords:** Modulation histogram, Coding map, Segmentation, Preprocessing, Binary defocusing, Digital fringe projection

## Abstract

Shadow and background are two common factors in digital fringe projection, which lead to ambiguity in three-dimensional measurement and thereby need to be seriously considered. Preprocessing is often needed to segment the object from invalid points. The existing segmentation approaches based on modulation normally perform well in pure dark background circumstances, which, however, lose accuracy in situations of white or complex background. In this paper, an accurate shadow and background removal technique is proposed, which segments the shadow by one threshold from modulation histogram and segments the background by the threshold in intensity histogram. Experiments are well designed and conducted to verify the effectiveness and reliability of the proposed method.

## Background

Digital fringe projection (DFP) techniques are widely employed in flexible, non-contact and high-speed 3D shape measurement [1]. In a DFP system, a sequence of phase-shifted sinusoidal fringes is often projected on the object by the projector, and the fringes are distorted by the object surface and captured by a camera. Phase map can be retrieved from the deformed fringes, and the object height information is calculated from the phase map in a calibrated DFP system [2]. However, shadow and the background are inevitable, since the projector and camera are arranged from different viewpoints. Invalid points such as shadow and background should be identified and removed from the object.

Researchers made great efforts to remedy the influence of invalid points including the shadow and background. Skydan et al. [3] utilized multiple projectors to probe the object from different viewpoints to achieve shadow free reconstruction. However, the increased cost of hardware keeps this method from commonly utilized. Zhang [4] proposed to employ the Gaussian filter on the fringes to remove random noise and identify the invalid points by the monotonicity of the unwrapped phase. However, the Gaussian filter introduces errors to the object details. Chen et al. [5] applied a threshold to the least-squares fitting errors in temporal phase unwrapping for invalid points detection. However, this method is vulnerable to noise [6].

Huang and Asundi [6] proposed a compact framework combining modulation, rms error and monotonicity for shadow and background removal and error detection. Intensity modulation is very effective in measuring how informative are the pixels, and can be used to detect background and shadow. However, manually adjusting the threshold is time-consuming. In practice, the threshold selection is subject to measurement conditions such as the environmental illumination and object surface characteristics. Lu et al. [7] proposed a technique to remove shadow points by mapping the 3D results into projector coordinates, and the modulation is not needed. However, this method can only detect shadow caused by the DFP system [8].

**Fig. 1:**
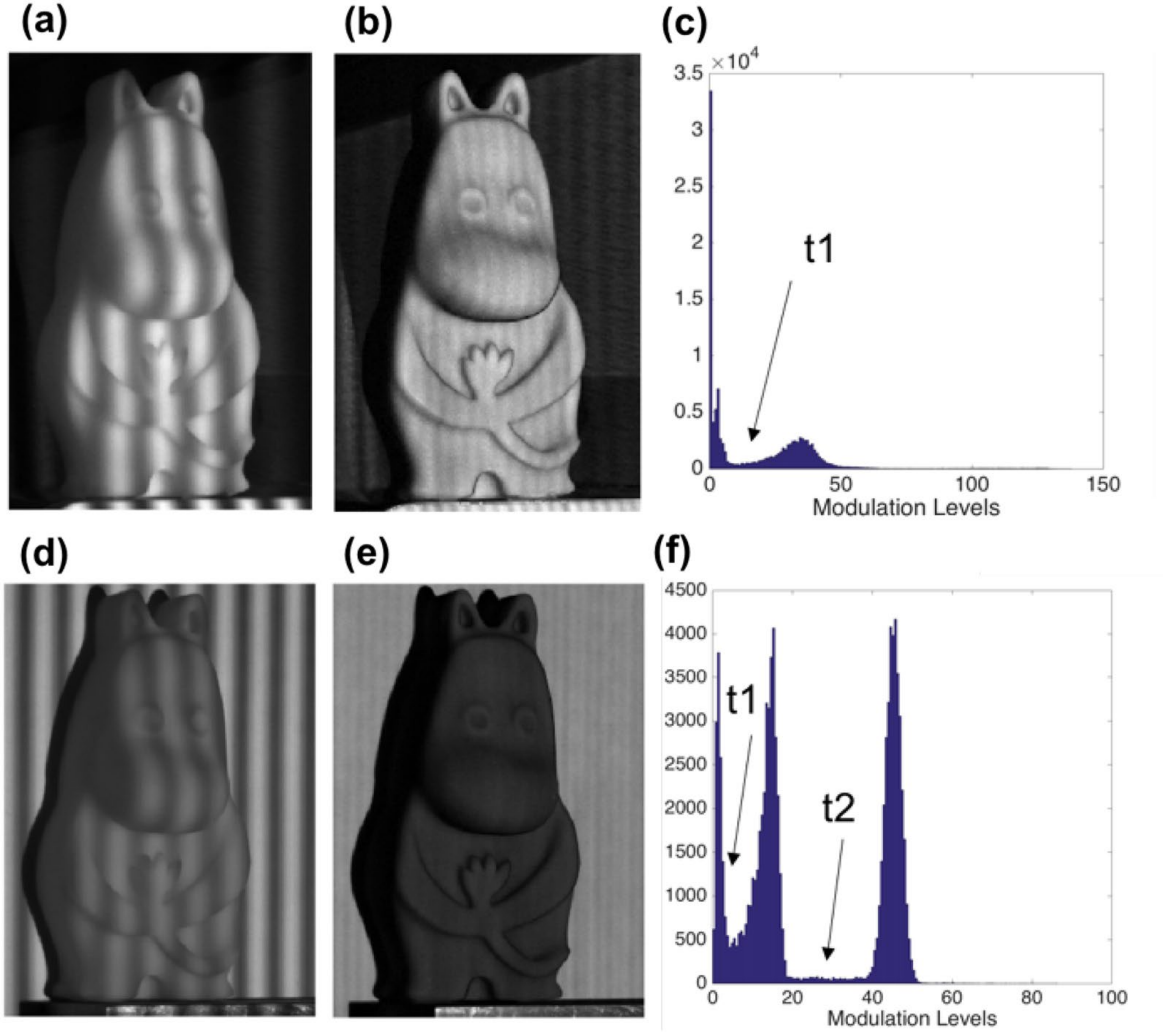
Comparison between dark background and white background. **a** One of captured fringes on the object with dark background, **b** modulation distribution of the captured fringes, **c** modulation histogram, **d** one of the captured fringes on the object with a white background, **e** modulation distribution of the captured fringes, **f** modulation histogram. In **f**, the lower threshold *t*_1_ is for shadow and interior invalid points detection, and the upper threshold *t*_2_ is for background detection

Otsu’s method [9] is widely utilized for thresholding in image segmentation, which is automatic and efficient. However, it fails to provide optimal threshold when the class to be separated increases or when the intensity histogram is close to unimodal distribution [10]. Ng [10] improved this technique through a weighting factor, considering the occurrence probability of the threshold point. Both Otsu’s method and Ng’s method aim for image segmentation based on intensity histogram. The literature [8] utilized the automatic thresholding method in modulation histogram for object detection. However, their method can only deal with dark background with low modulation, since the background and shadow are with similar low modulation, while the object is with obviously higher modulation level, and only one threshold is needed to segment the object. When the background is a white board or complex with higher or similar modulation level, it is difficult to segment the background from the object. In this situation, there will be three classes in the modulation map, and two thresholds are needed to separate the object from the background and shadow, as shown in Fig. 1. The method in [8] cannot deal well with this situation.

In this paper, we apply the multi-thresholding technique on modulation histogram and propose a preprocessing method to detect the valid points of the object by firstly segmenting the shadow using one threshold from the modulation histogram. Secondly, we project one more picture onto the object and reference plane and calculate the intensity difference of the captured images, and the histogram of the difference map is analyzed for the background detection. We call this one more picture the coding map.

The rest of this paper is organized as follows: We introduce the related principles and existing methods in Related work. In “Methods” section, we introduce the details of how to implement our proposed object segmentation technique. In the experiments and results part, we present and compare some segmentation results using our method and the expanded conventional method. The 3D shape reconstruction result is also presented in this section. In the end, we make a summary in “Conclusion”.

## Related work

### N-step phase shifting and modulation

Phase-shifting algorithms are widely utilized in the stationary object measurement due to their high accuracy and flexibility [11]. They carry out point-by-point measurement and calculate wrapped phase value from −*π* to *π*. For the N-step phase-shifting method, sinusoidal fringes with the following intensity modulation are often used [4],

1 $$I_{n} \left( {x,y} \right) = I_{\text{a}} + I_{\text{m}} \cos \left[ {\varphi \left( {x,y} \right) + \frac{{2\pi \left( {n - 1} \right)}}{N}} \right]$$

where *n* is the phase-shifting number and *N* is the total phase-shifting steps. *I*_*n*_ is the intensity map of the *n*th sinusoidal fringes and *I*_a_ and *I*_m_ are the average intensity and modulation intensity, respectively. The wrapped phase *φ* ^w^ can be calculated as [6],

2 $$\varphi^{\text{w}} = - \tan^{ - 1} \frac{{\mathop \sum \nolimits_{n = 0}^{N - 1} I_{n} \cdot \sin \frac{2n\pi }{N}}}{{\mathop \sum \nolimits_{n = 0}^{N - 1} I_{n} \cdot \cos \frac{2n\pi }{N}}}$$

The modulation *M* is defined as,

3 $$M = \frac{2}{N}\sqrt {\left[ {\mathop \sum \limits_{n = 0}^{N - 1} I_{n} \cdot \sin \frac{2n\pi }{N}} \right]^{2} + \left[ {\mathop \sum \limits_{n = 0}^{N - 1} I_{n} \cdot \cos \frac{2n\pi }{N}} \right]^{2} }$$

It shows how much useful information is contained in each pixel. It is usually selected as the reliability map to guide the phase unwrapping and object segmentation [12]. If the proper threshold *t* is found, object can be identified from the background, shadow and the less informative pixels. However, manually adjusting the modulation threshold is very tedious and unstable, since the modulation varies according to measuring conditions, such as the incoherent light, the reflection of object and background, and the occlusion caused by object step height.

### Existing methods of threshold selection

Otsu’s method is commonly utilized for quick segment of the object and background based on image intensity. For a given image, if we distribute the gray levels into *L* bins ranging from 1 to *L*, *k*_*i*_ represent the total number of pixels with gray-level *i* and *K* is the total pixels of the given image, $K = k_{1} + k_{2} + \cdots + k_{\text{L}}$. The occurrence probability of gray-level *i* is calculated as,

4 $$p_{i} = \frac{{k_{i} }}{K},\quad p_{i} \ge 0, \quad \mathop \sum \limits_{i = 1}^{L} p_{i} = 1.$$

When a single value threshold is applied, the pixels of the given image are to be divided into two classes (typically the object and background with shadow): class *C*_0_ includes the pixels with levels $\left\{ {k_{1} ,k_{2} , \ldots ,k_{t} } \right\}$, and class *C*_1_ includes the pixels with levels $\left\{ {k_{t + 1} ,k_{t + 2} , \ldots ,k_{L} } \right\}$, where *k*_*t*_ is the threshold to be determined. The occurrence probability of each class can be calculated as,

5 $$\omega_{0} = P_{r} \left( {C_{0} } \right) = \mathop \sum \limits_{i = 1}^{t} p_{i} = \omega \left( t \right)$$

6 $$\omega_{1} = P_{r} \left( {C_{1} } \right) = \mathop \sum \limits_{i = t + 1}^{L} p_{i} = 1 - \omega \left( t \right)$$

and the class mean levels are,

7 $$\mu_{0} = \mathop \sum \limits_{i = 1}^{t} i \cdot p_{i} /\omega_{0} = \mu \left( t \right)/\omega \left( t \right)$$

8 $$\mu_{1} = \mathop \sum \limits_{i = t + 1}^{L} i \cdot p_{i} /\omega_{1} = \frac{{\mu_{\varGamma } - \mu \left( t \right)}}{1 - \omega \left( t \right)}$$

where *ω*(*t*) and *μ*(*t*) are the zeroth-order and the first-order cumulative moments of the histogram up to *t*th level, respectively. The total average gray level of the whole image is calculated as,

9 $$\mu_{\varGamma } = \mathop \sum \limits_{i = 1}^{L} i \cdot p_{i}$$

For any selection of *t*, it is easily verified that

10 $$\omega_{0} \cdot \mu_{0} + \omega_{1} \cdot \mu_{1} = \mu_{\varGamma }$$

11 $$\omega_{0} + \omega_{1} = 1$$

According to the discriminant criterion analysis [9], Otsu showed that the optimal threshold $t^{*}$ can be calculated by maximizing the between-class variance,

12 $$t^{*} = {\text{Arg}}\,{\text{Max}}\left\{ {\sigma_{B}^{2} \left( t \right)} \right\}$$

where the between-class variance $\sigma_{\text{B}}^{2}$ is defined as,

13 $$\sigma_{\text{B}}^{2} = \omega_{0} \left( {\mu_{0} - \mu_{\varGamma } } \right)^{2} + \omega_{1} \left( {\mu_{1} - \mu_{\varGamma } } \right)^{2}$$

The optimal threshold $t^{*}$ is often calculated by an equivalent, but simpler equation [13],

14 $$t^{*} = {\text{Arg}}\,{\text{Max}}\left\{ {\omega_{0} \mu_{0}^{2} + \omega_{1} \mu_{1}^{2} } \right\}$$

Otsu’s method works well on the histogram of bimodal distribution, but not robust for histograms of unimodal or close to unimodal [10]. Ng [10] developed a valley emphasis method to improve Otsu’s method. By adding a weighting factor, then the threshold is calculated by considering two elements, the small occurrence and the big between-class variance. The threshold of Ng’s method is calculated as,

15 $$t_{\text{v}}^{*} = {\text{Arg}}\,{\text{Max}}\left\{ {\left( {1 - p_{t} } \right)\sigma_{\text{B}}^{2} \left( t \right)} \right\}$$

The above two methods for automatic threshold selection are intended for image segment based on gray-level histogram. The literature [8] utilizes them in modulation histogram for object segmentation. However, in their work, the background is dark, so invalid points in shadow and background are with low modulation level, and the object is with higher modulation level; only one threshold is enough to segment the object. As shown in Fig. 1, Fig. 1a shows a captured fringe on the object with dark background, Fig. 1b shows the modulation map of the captured fringes, and Fig. 1c shows the histogram of the modulation map. The modulation histogram is within two classes, and it is easy to find the threshold *t*_1_, to segment the valid points and invalid points.

In practical, the modulation histogram is not necessarily in two classes, such as when a white board is used as the background for system calibration, as shown in Fig. 1d. Figure 1e shows the modulation map of Fig. 1d, and Fig. 1f shows the histogram of the modulation map. As can be seen that when the background is a white board, the modulation level of the background will be high, and the modulation histogram in Fig. 1f is to be classified to three categories. The background is with middle to high modulation, the object is with medium modulation, and the shadow is with low modulation level. Two thresholds need to be calculated for shadow and the background segmentation separately. For this situation, the conventional method cannot be utilized directly.

## Methods

**Fig. 2:**
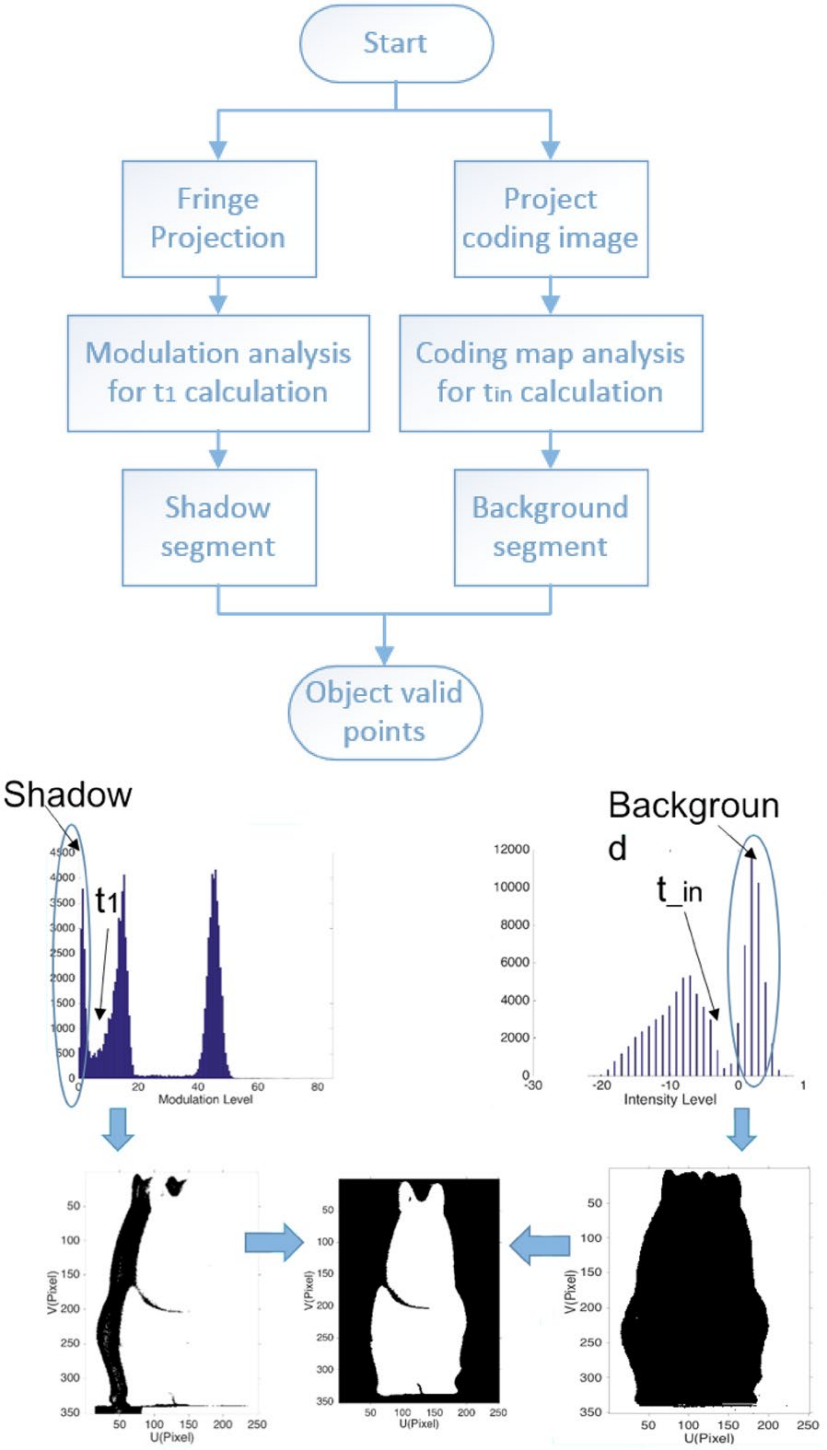
Flowchart of object valid points detection

To segment the object from white background, or complex background, we firstly applied the expanded Ng’s method for multi-threshold calculation in modulation histogram. Then, we proposed our method for shadow and background detection. Figure 2 shows the flowchart of our method. The first threshold calculated from modulation histogram is utilized for shadow segmentation. For the background segmentation, we project one coding image onto the object and calculate the intensity difference between the object and the background. The threshold in intensity histogram is used for background segmentation. Details on how to segment the shadow and background are introduced as follows.

### Expanded thresholding method

The literature [8] has improved and applied Ng’s method for single thresholding in the fringe modulation histogram for object detection in digital fringe projection technique, while it only discussed the situation of a dark background, in which only one threshold is needed for object segmentation. For DFP system with a white or complex background, we apply the multi-thresholding Ng’s method on the modulation. The expanded Ng’s method can be described by [9],

16 $$\left\{ {t_{1}^{*} ,t_{2}^{*} , \ldots t_{M - 1}^{*} } \right\} = {\text{Arg }}\,{\text{Max}}\left\{ {\left( {1 - \mathop \sum \limits_{j = 1}^{M - 1} p_{tj} } \right)\left( {\mathop \sum \limits_{k = 1}^{M} \omega_{k} \cdot \mu_{k}^{2} } \right)} \right\}$$

**Fig. 3:**
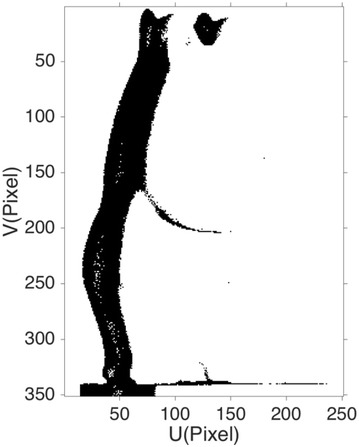
Detected shadow and interior invalid points using threshold *t*_1_ on the modulation histogram

Utilizing this equation, two thresholds *t*_1_ and *t*_2_ in Fig. 1f can be calculated. Pixels with modulation level smaller than *t*_1_ are regarded as the shadow, pixels with modulation level larger than *t*_2_ are regarded as background, and the object pixels are with medium modulation level. However, the multi-threshold calculation is less credible [9]. What’s worse, when the background is complex, with modulation levels distributed for a large range, it is difficult to segment the background by just modulation. In our method, only *t*_1_ is utilized for shadow detection, and the background is segmented from image intensity. Figure 3 shows the preliminary detection results, and black pixels are shadow and invalid points.

### Intensity-based background segmentation

For background segment, we project an extra coding image with intensity of Eq. (17) on the object and background and analyze the intensity of their difference to calculate a reliable *t*_in_.

17 $$I\left( {x,y} \right) = 255 \times \frac{x}{N}$$

**Fig. 4:**
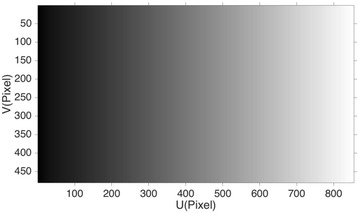
The intensity distribution of the coding image: it would be projected on the object and reference plane, and the difference of the captured images are used for calculating the threshold for background detection

**Fig. 5:**
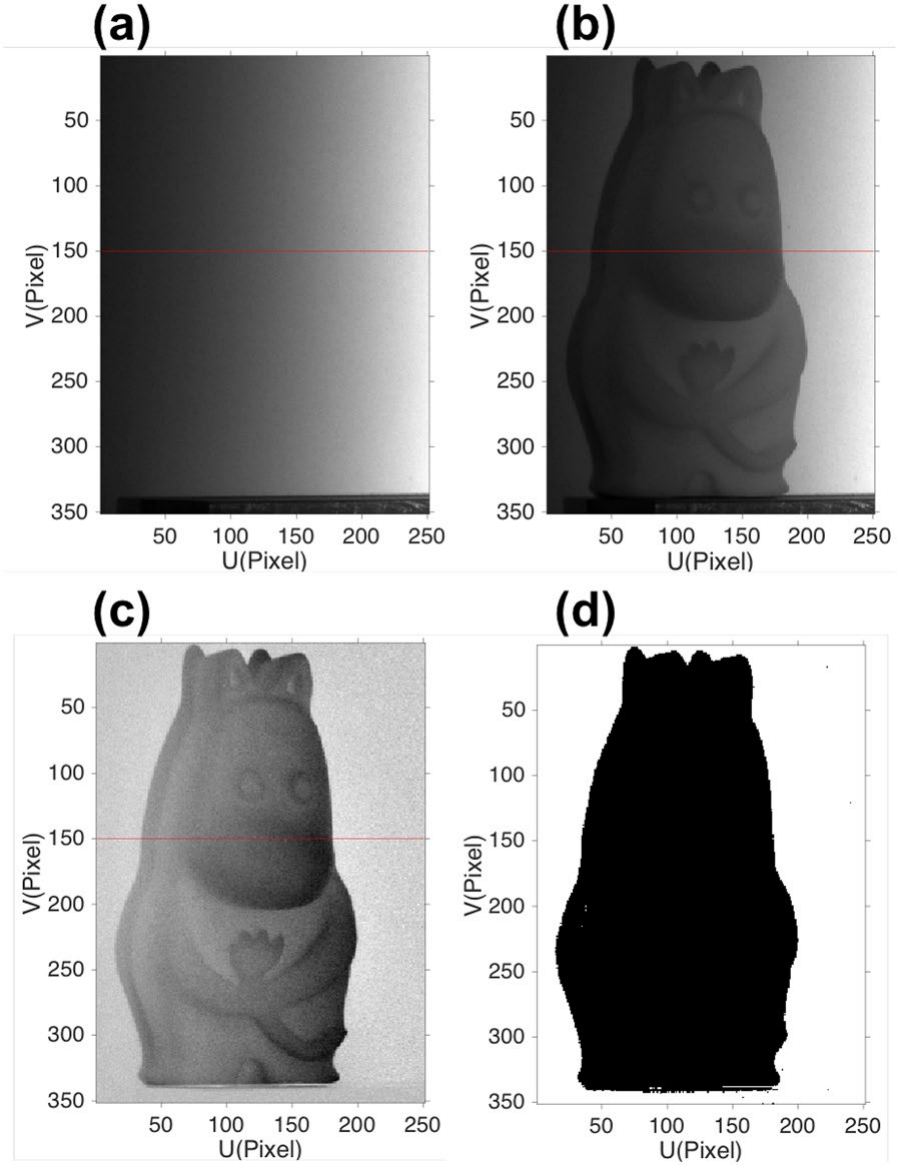
The captured coding images on **a** the reference plane; **b** the object; **c** the intensity difference map between **a** and **b**; **d** by binarizing **c**, we can segment the background from the object with shadow. Cross-section intensity at the red line position showing in **a**–**c** is analyzed in Fig. 6b

Here 255 is the total gray-level range, and *N* is the column of the projected image. The coding image for projection is shown in Fig. 4. The captured coding image on the reference plane *I*_flat_ is shown in Fig. 5a, and the captured coding image on the object *I*_obj_ is shown in Fig. 5b. The intensity difference map *I*_diff_ shown in Fig. 5c is calculated by subtracting *I*_flat_ from *I*_obj_. Here (*x*, *y*) is omitted for simplicity.

18 $$I_{\text{obj}} - I_{\text{flat}} = I_{\text{diff}}$$

Since the extra projected image contains a lot of useful information for background detection, we call it the coding map.

**Fig. 6:**
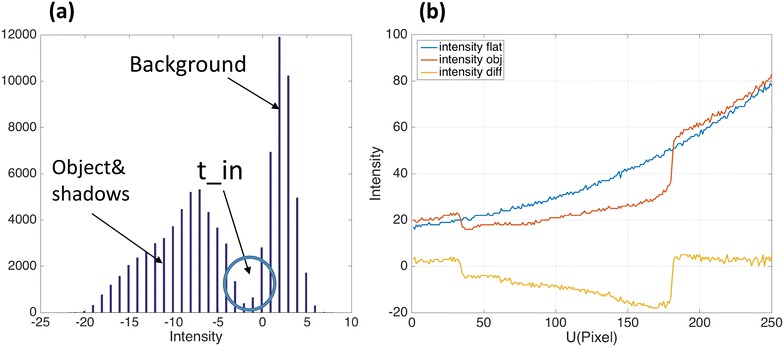
**a** The histogram of Fig. 5c, according to which the threshold *t*_in_ is calculated. Pixels with intensity difference larger than the threshold are regarded as the background, and the pixels with intensity difference smaller than the threshold are the object and shadow. **b** The 150th row cross-section intensity of Fig. 5a–c

The histogram of difference coding map *I*_diff_ is shown in Fig. 6a. Utilizing the single threshold criteria in [10], we can calculate a reliable intensity threshold *I*_in_ for segmenting the background. The 150th row cross-section intensity of Fig. 5a–c is shown in Fig. 6b.

So with the multi-thresholding Ng’s method utilized on modulation histogram, the object valid points matrix *V*_valid_ is computed as,

19 $$V_{\text{valid}} = B\left( {M,t_{1} } \right) \circ \neg B\left( {M,t_{2} } \right)$$

where B is a matrix with the same size as M, calculated as, $$B_{ij} \left( {M,t} \right) = \left\{ {\begin{array}{*{20}l} {1,} \hfill & {{\text{where}}\quad M_{ij} > t} \hfill \\ 0 \hfill & {{\text{where}}\quad M_{ij} \le t} \hfill \\ \end{array} } \right.,\quad M$$, is the matrix of modulation map and *t*_1_ and *t*_2_ are the first and second threshold of modulation histogram calculated by (16). ° represents the Hadamard product of two matrices, and $\neg$ means negative. Multi-threshold calculation is less credible [9], and the background may be complex. We analyze intensity difference of the coding map to find *t*_in_ for background segmentation, and the lower threshold *t*_1_ from modulation is still used for shadow detection. The proposed object valid points matrix *V*_pro_ is calculated as,

20 $$V_{\text{pro}} = B\left( {M,t_{1} } \right) \circ \neg B\left( {I_{\text{diff}} ,t_{\text{in}} } \right)$$

where *I*_diff_ is the intensity difference map calculated from Eq. (18) and *t*_in_ is the intensity threshold.

## Experiments and results

**Fig. 7:**
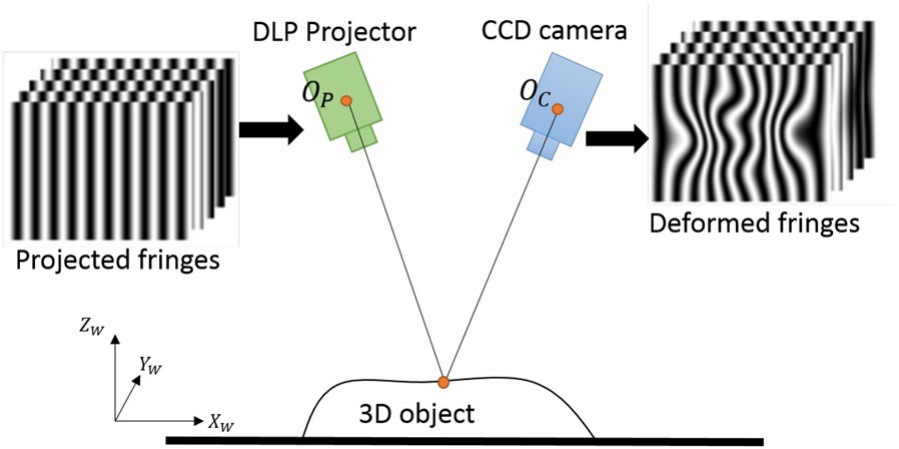
3D shape measurement system

Experiments are carried out to test the proposed shadow and background removal technique. A DFP 3D shape measurement system in Fig. 7 with defocused projector projecting binary fringes of width *T* = 30 is employed to measure the 3D objects. Utilizing defocused binary fringes can avoid nonlinear gamma correction [14]. The projected fringes are deformed by the object and captured by a camera. Phase of the object surface is retrieved by phase-shifting technique, and height information is calculated after system calibration [15]. The hardware in the study includes a DLP projector of model AAXA P4-X with native resolution of 480 × 854 pixels and a CCD camera of Point Gray FL3-U3-13S2M-CS with resolution of 1328 × 1048 pixels. The camera is attached with a 6-mm focal-length lens of model Kowa LM6JC. The projection distance is about 40 cm.

### Shadow and background segmentation

**Fig. 8:**
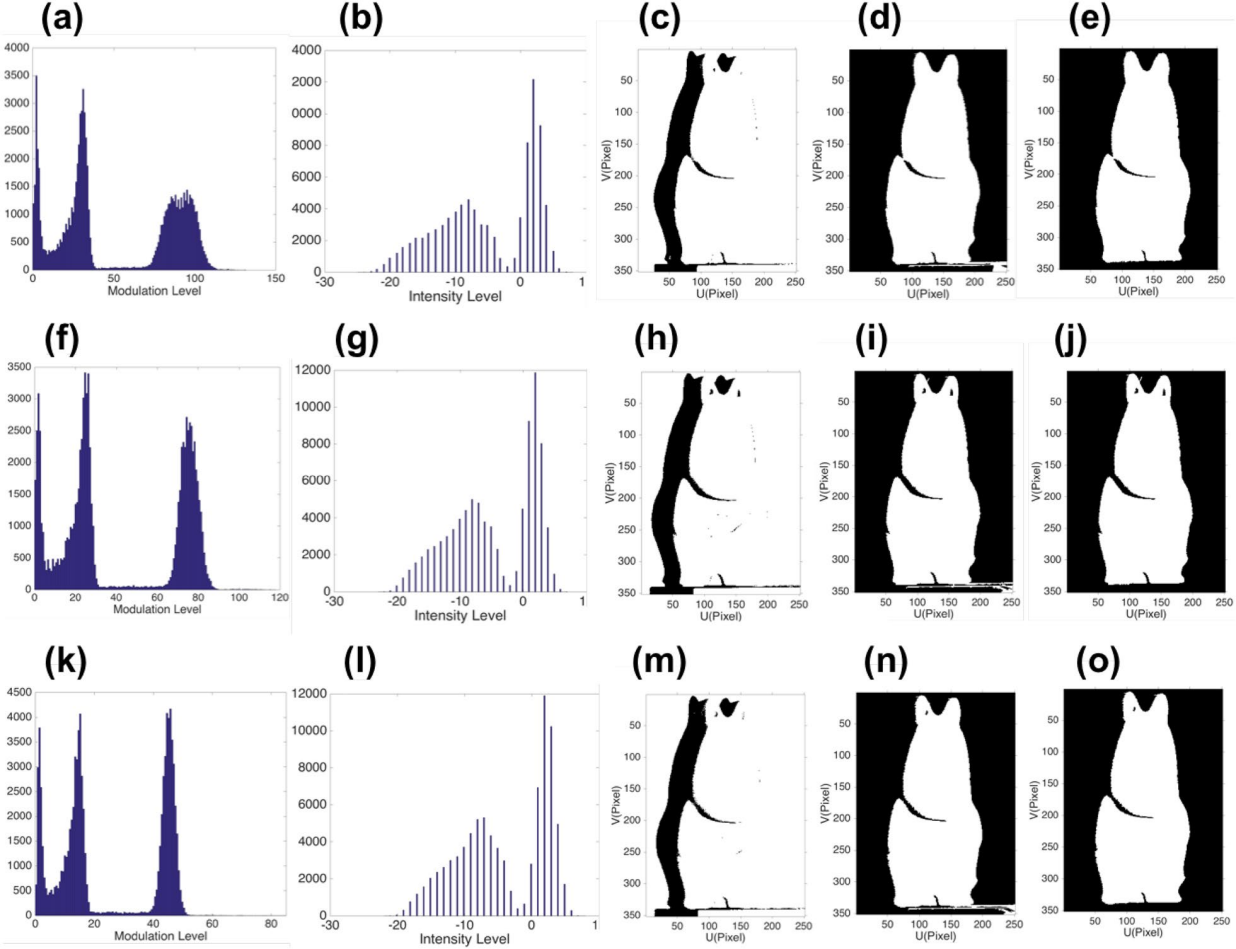
Shadow and background removal comparison 1. **a** The modulation histogram, **b** the intensity difference histogram, **c** object segmented by single threshold *t*_1_, **d** object segmented by *t*_1_ and *t*_2_ based on modulation, **e** object segmented by *t*_1_ from modulation and *t*_in_ from intensity difference histogram when utilizing a nearly focused projector; slightly defocused projector (**f**)–(**j**); and strongly defocused projector (**k**)–(**o**)

**Fig. 9:**
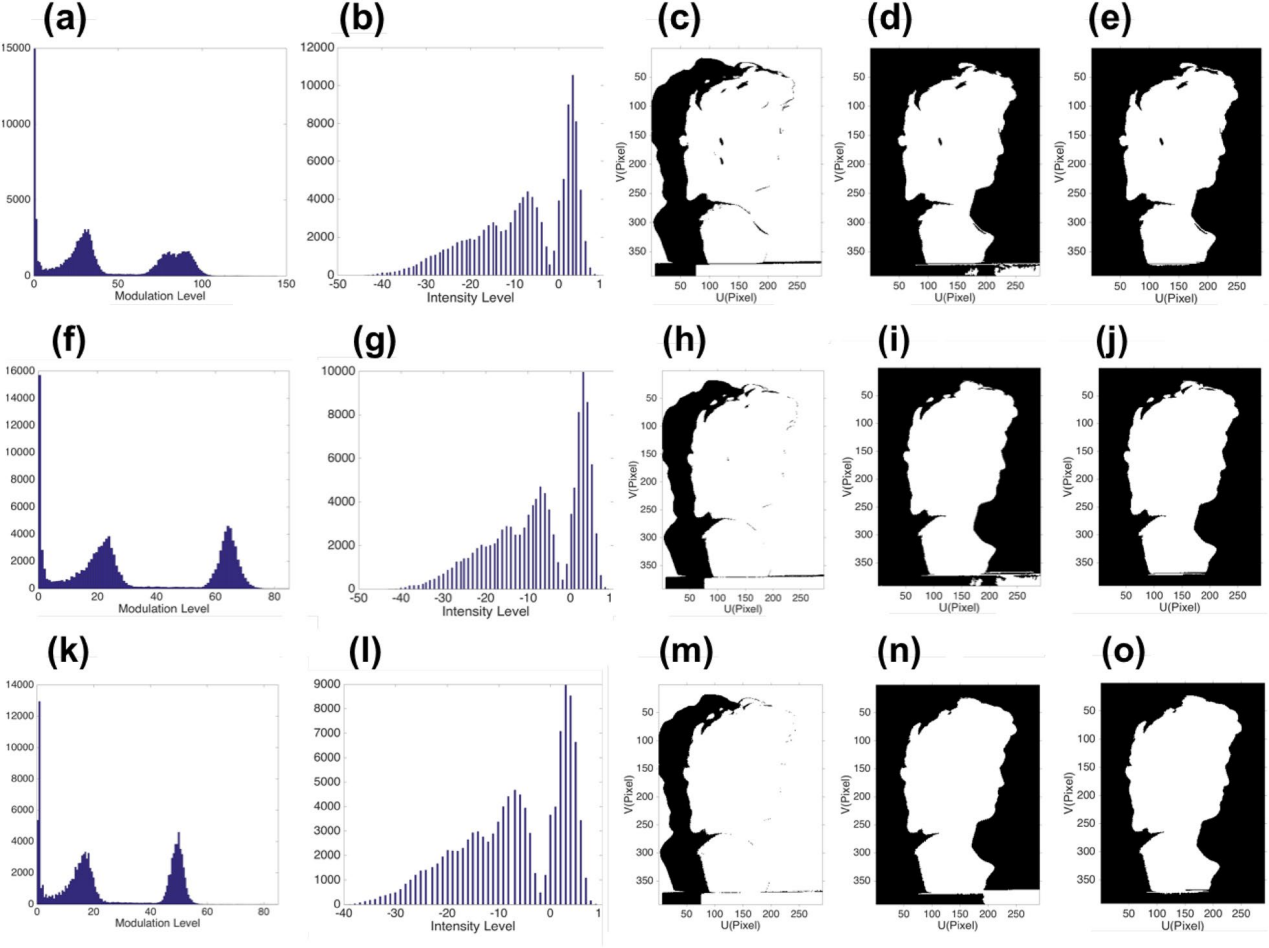
Shadow and background removal comparison 2. **a** The modulation histogram, **b** the intensity difference histogram, **c** object segmented by single threshold *t*_1_, **d** object segmented by *t*_1_ and *t*_2_ based on modulation, **e** object segment by *t*_1_ from modulation and *t*_in_ from intensity difference histogram when utilizing a nearly focused projector; slightly defocused projector (**f**)–(**j**); and strongly defocused projector (**k**)–(**o**)

**Fig. 10:**
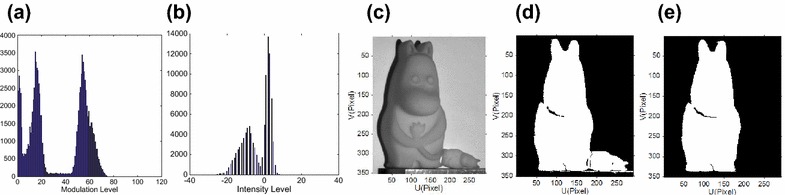
Object segmentation with a complex background. **a** The modulation histogram, **b** the histogram of the intensity difference for the captured coding map, **c** the object on the left side, and the smaller one beside needs to be segmented as the background, **d** segmentation result by modulation only, **e** segmentation result by our method

In this experiment, two different objects are tested and segmented, and the results are shown in Fig. 8 for the first object and Fig. 9 for the second object. The calculated thresholds are shown in Table 1. Three different defocusing levels of the projector are utilized, to produce different fringe contrasts and modulation levels. Figure 8a shows the modulation histogram of the captured fringe patterns, and Fig. 8b shows the histogram of intensity difference for the captured coding image. Figure 8c shows the object segmentation by single threshold, as we can see from this picture, only one threshold is not enough to segment the whole object when the background is with high modulation level. It only segments the shadow from the object. Figure 8d shows the detected object by modulation thresholds *t*_1_ and *t*_2_, as we can see, it can segment the shadow and background from the object, but part of the background is detected as the valid points of the object. There are two reasons: First, multi-threshold calculation is not always credible [9], and second, when the background is complicated with modulation levels distributed in both the second cluster and the third cluster, background segmentation based on pure modulation is prone to error. Figure 8e shows the detected object by our proposed method, the background is segmented based on the intensity difference histogram of the coding map shown in Fig. 8b, and threshold *t*_in_ is utilized. We may notice that the detected object is more accurate than Fig. 8c. The similar trends are shown in Fig. 8f–j for slightly defocused projector and Fig. 8k–o for strongly defocused projector. They provide different fringe contrasts and modulation levels. We may see that when the projector defocusing level increases, the modulation thresholds *t*_1_ and *t*_2_ become smaller, because the defocusing will depress the fringe modulation level in general. The same experiments are also done on the second object, and similar results are shown in Fig. 9. To demonstrate that our proposed method can work with a more complex background, we put a small statue near the measuring object to make the background more complex. Results are shown in Fig. 10. Figure 10a shows the modulation histogram of the captured fringes, Fig. 10b shows the histogram of the intensity difference for the captured coding map, and Fig. 10c shows the object with a small statue beside it. Object segmented by Ng’s method based on modulation is shown in Fig. 10d, and by our proposed method, it is shown in Fig. 10e. We may see that our proposed method can accurately segment the object from background, while the modulation-based method cannot segment the object from complex background. Our proposed method can segment valid points of the object more accurately than that of pure modulation, in most practical conditions.

**Table 1:** Modulation and intensity thresholds calculated for two objects with different projector defocusing levels

Object	Projector defocusing conditions	Modulation thresholds		Intensity threshold
		*t*_1_	*t*_2_	*t*_in_
Object1	Nearly focused	8	53	− 1.5
	Slightly defocused	8	44	− 2.5
	Strongly defocused	4	22	− 2.5
Object2	Nearly focused	15	49	− 1.5
	Slightly defocused	7	37	− 3.5
	Strongly defocused	7	25	− 3.5

### 3D reconstruction

After we retrieved the phase map of the object, the height information can be calculated by system calibration [15]. One commonly utilized method calibrates the camera and the projector separately to find the system parameters [16]. This kind of method is easy to understand, because each system parameter has its geometric meaning, but is also time-consuming, and error prone [17]. Because the projector is regarded as an inversed camera, its calibration accuracy depends on the camera calibration process. In this work, we apply the calibration framework presented in [15] to calculate the height information of the object.

For a general DFP system with arbitrary arrangements, the governing equation of the 3D height is computed as [18, 19],

21 $$\begin{aligned} z & = f_{c} /f_{d} , \\ f_{c} & = 1 + c_{1} \varphi + \left( {c_{2} + c_{3} \varphi } \right)i + \left( {c_{4} + c_{5} \varphi } \right)j \\ & \quad + \left( {c_{6} + c_{7} \varphi } \right)i^{2} + (c_{8} + c_{9} \varphi )j^{2} , \\ f_{d} & = d_{0} + d_{1} \varphi + \left( {d_{2} + d_{3} \varphi } \right)i + \left( {d_{4} + d_{5} \varphi } \right)j \\ & \quad + \left( {d_{6} + d_{7} \varphi } \right)i^{2} + (d_{8} + d_{9} \varphi )j^{2} , \\ \end{aligned}$$

where *z* is the height at pixel (*i*, *j*) and *φ* is the phase value of the projection fringe at that pixel. *c*_1_–*c*_9_ and *d*_0_–*d*_9_ are constants related to system parameters. To determine the 19 coefficients, we need to know some sample points height information on the calibration board, their corresponding phase *φ* and pixel position (*i*, *j*) and use least-squares algorithm to find the coefficients.

In our experiment, a 2D checkerboard with 12 × 16 black and white squares is utilized as the calibration object. The calibration includes obtaining the 3D coordinates and phase value of all calibration points on the checkerboard, at ten different positions. Phase-shifted sinusoidal fringes and an extra white image are projected on to the calibration board and captured by the camera. The camera intrinsic and extrinsic parameters are calibrated with the captured clear checkerboard. We define the points in the world and camera coordinate system as $\left\{ {x_{\text{w}} , \left. {y_{\text{w}} , z_{\text{w}} } \right\}} \right.^{\text{T}}$ and $\left\{ {x_{\text{c}} , \left. {y_{\text{c}} , z_{\text{c}} } \right\}} \right.^{\text{T}}$, respectively. Generally, *z*_w_ is set to zero, so the relationship between the world and camera coordinate systems is expressed by,

22 $$\left\{ {\left. {\begin{array}{*{20}c} {x_{\text{c}} } \\ {y_{\text{c}} } \\ {z_{\text{c}} } \\ \end{array} } \right\} = \left[ {\begin{array}{*{20}c} {R_{11} } & {R_{12} } & {T_{1} } \\ {R_{21} } & {R_{22} } & {T_{2} } \\ {R_{31} } & {R_{32} } & {T_{3} } \\ \end{array} } \right]} \right.\left\{ {\left. {\begin{array}{*{20}c} {x_{\text{w}} } \\ {y_{\text{w}} } \\ 1 \\ \end{array} } \right\}} \right.,$$

here R and T represent the rotation and translation elements of the camera extrinsic parameters. Using Eq. (22), we can find all the calibration points in the camera coordinate system. Set the first calibration board position as the reference plane and its coordinate system as the world coordinate system. The literature [15] computes the reference plane equation in camera coordinate system and calculates the distance of each calibration point to this plane as the points’ height. In our experiments, all the calibration points are transformed to the world coordinate system according to their respective transformation matrix; then, *Zw* is the point’s height.

The system coefficients *c*_1_–*c*_9_ and *d*_0_–*d*_9_ are computed through minimizing a nonlinear least-squares error function as,

23 $$\arg \mathop {\hbox{min} }\limits_{c,d} \mathop \sum \limits_{k = 1}^{m} \left( {\frac{{f_{c} }}{{f_{d} }} - z_{k}^{b} } \right)^{2} ,$$

where *k* is the ordinal number of each point and m denotes the total number of points. An initial guess of coefficients *c*_1_–*c*_9_ and *d*_0_–*d*_9_ is obtained by minimizing a linear least-squares error of $S = \mathop \sum \limits_{k = 1}^{m} \left( {f_{c} - f_{d} z_{k}^{b} } \right)^{2}$; then, Levenberg–Marquardt algorithm is utilized to verify the results.

**Fig. 11:**
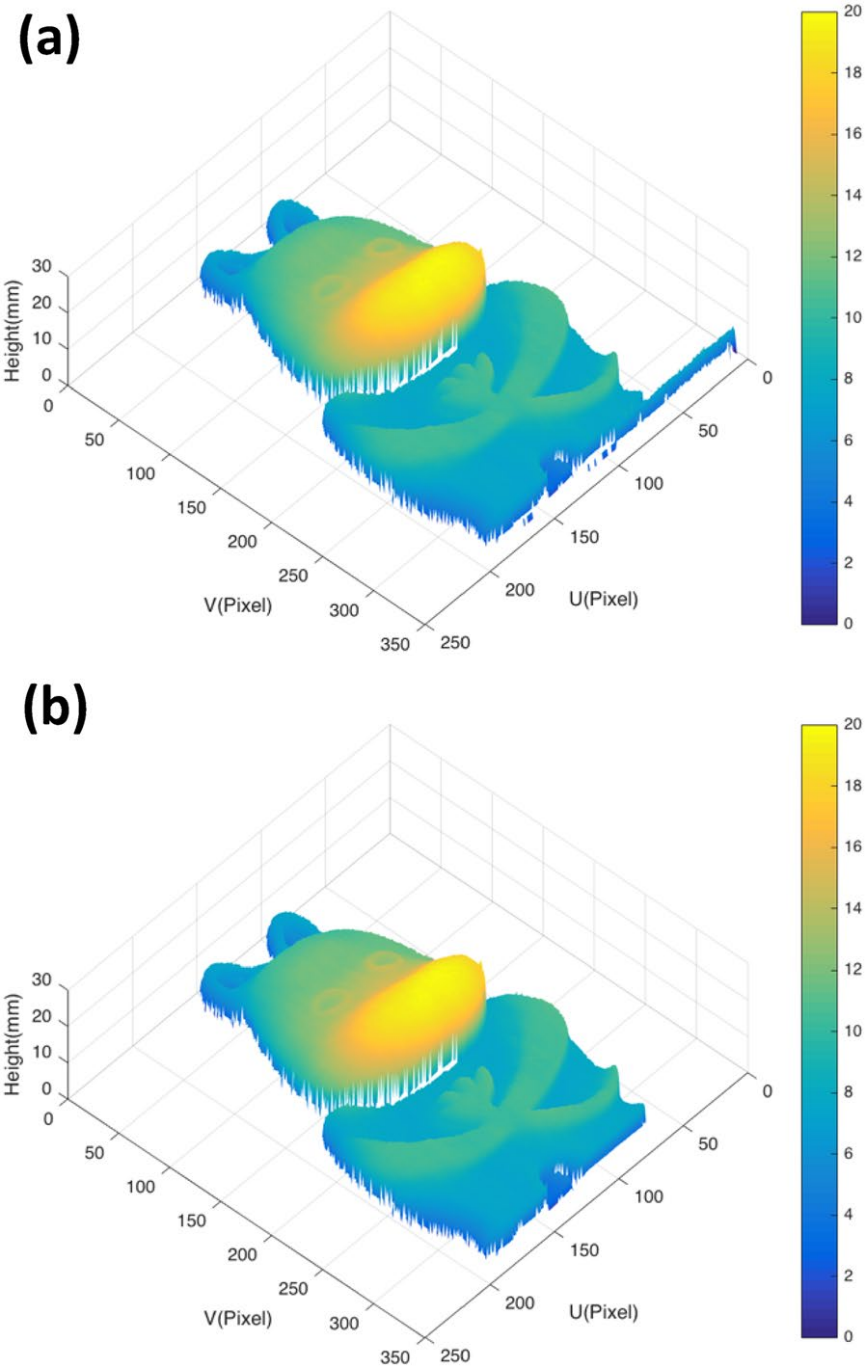
3D measurement results with the object segmented by multi-thresholding Ng’s method on modulation histogram (**a**) and by our proposed method (**b**)

The reconstructed 3D object is shown in Fig. 11. The object in Fig. 11a is preprocessed by object segmentation based on modulation histogram, and that of Fig. 11b is preprocessed by our proposed method with modulation and intensity histogram being analyzed. As we can see, the modulation-based segmentation can remove the shadow correctly, so as our proposed method. However, in Fig. 11a, part of the measurement platform is segmented as part of the object, which should be removed as background, while our proposed method can accurately remove the shadow and complex background from the object points.

## Conclusion

In this paper, we proposed a novel preprocessing method for object segmentation in DFP 3D shape measurement. We firstly applied the multi-threshold Ng’s method on modulation histogram and then proposed our method for shadow and background detection based on modulation and intensity histogram. Experiments verified that our proposed method can improve the 3D shape measurement with white and complex background.
